# The Role of Formyl Peptide Receptor 1 Gene Polymorphisms in Human Colorectal Cancer

**DOI:** 10.7150/jca.36355

**Published:** 2020-03-25

**Authors:** Shu-Qin Li, Yang Yu, Yan Zhang, Yan-Ping Sun, Xin-xing Li, Ning Su

**Affiliations:** 1 School of Pharmacy, Shanghai Jiao Tong University, 800 Dongchuan Road, Shanghai, 200240, China.; 2 School of Pharmacy, Ruijin Hospital Affiliated to Shanghai Jiao Tong University School of Medicine, 197 Ruijin Road (No.2), Shanghai, 200025, China.; 3 Department of General Surgery, Shanghai Chang Zheng Hospital, Second Military Medical University, 415 Fengyang Road, Shanghai, 200003, China.

**Keywords:** Colorectal cancer, Formyl peptide receptor 1, Single nucleotide polymorphisms, Metastasis

## Abstract

Formyl peptide receptor 1 (FPR1) belongs to G protein-coupled receptors expressed mainly in phagocytic leukocytes. The gene encoding FPR1 is highly polymorphic and related to inflammation. In this study, we investigated the single nucleotide polymorphisms (SNPs) of Fpr1 in human colorectal cancer (CRC), and analyzed the association of Fpr1 SNPs with clinicopathological parameters and some specific diagnostic markers of CRC. Although the allele and genotype frequencies of Fpr1 SNPs in CRC tissues were not significantly different from that in whole blood cells derived from healthy Chinese subjects. Significant associations were observed between genotypes of c.289C>A and distant metastasis (P=0.001), and between genotypes of c.306T>C and tumor size (P=0.016). Genotypes of c.546C>A was closer to tumor size and lymphatic invasion (P=0.012 and P=0.043, respectively). Meanwhile, genotypes of c.1037C>A was related with tumor location and differentiation (P=0.000 and P=0.005, respectively). Besides, genotypes of c.576T>C>G was related with pathological type (P=0.000). Furthermore, several Fpr1 SNP positions including c.289 (C>A) and c.576 (G>C>T) were related to the expression of P53 (P=0.004 and P=0.008, respectively), and similar results were observed between other Fpr1 SNP positions and CEA, HER2 and Ki-67 (P<0.05). Our data demonstrate that Fpr1 SNPs may play the important role in the progression and metastasis of CRC.

## Introduction

Colorectal cancer (CRC) is one of the most frequently occurring malignancies in the world 1. In 2018, CRC accounted for more than 860,000 deaths with 1.8 million new cases 1. Several risk factors contribute to CRC progression, including advancing age, chronic intestinal inflammation, and genetic aberrations/mutations 2-4. Current evidence suggests that genetic contribution is about 30% 5. Multiple genetic studies have shown that the single nucleotide polymorphisms (SNPs) are associated with the occurrence, development, metastasis and drug tolerance of CRC 6,7.

The human formyl peptide receptor 1 (FPR1) belongs to the seven-transmembrane G protein-coupled receptors (GPCRs) 8. FPR1 is known to have important functions in host defense and inflammation. It is expressed mainly on phagocytic leukocytes, neutrophils, and monocytes 8. Binding of agonistic ligands to FPR1 results in activation of a G protein-mediated signaling cascade; leading to cell chemotaxis, calcium flux, phagocytosis, and release of inflammatory factors 8-10. FPR1 has also been detected on cells of non-hematopoietic tissues such as epithelial cells, endothelial cells, and neurons 11. Besides, recent evidence shows that it is expressed in several types of human cancer tissues such as gastric cancer 12,13, lung cancer 14, breast cancer 15, etc. Our previous study revealed the high expression of FPR1 in primary human CRC tissue 16. FPR1 mRNA expression was also associated with tumor serosal infiltration in CRC patients 16. Human *Fpr1* is a 6-kb single copy gene and located on chromosome 19 (19q13.3) 8. Several SNPs of *Fpr1* has been reported previously to be associated with blood pressure 17, C-reactive protein (CRP) level 18, E-selectin expression 19, and inflammatory disease such as aggressive peritonitis 20,21. In addition, *Fpr1* SNP (rs1042229) was also associated with stomach cancer 22. However, the function of *Fpr1* SNPs in human colorectal cancer has not been investigated.

In this study, we enrolled forty-eight Chinese CRC patients. Seven *Fpr1* SNPs (c.289C>A, c.301G>C, c.306T>C, c.546C>A, c.568A>T, c.576T>C>G, and c.1037C>A) were detected. The allele and genotype frequencies of these SNPs in CRC tissues were not significantly different from that in whole blood cells derived from healthy Chinese subjects. We further examined the correlation between *Fpr1* SNPs with clinicopathological characteristics and several specific diagnostic markers of CRC.

## Materials and methods

### Human subjects

A total of 60 surgically resected CRC tissue specimens were obtained from Changzheng Hospital, Shanghai, China. Histological classification and stage of the CRC were determined according to the UICC TNM classification system 23. Acquisition of tissue specimens was approved by the Changzheng Hospital Institution Review Board of and was carried out under guidance of established ethical protocols. Patients with colorectal cancer were between 22 and 83 years old, and colonoscopy and pathological examinations were confirmed. Exclusion criteria: (1) with a history of other tumor diseases, or with other genetic diseases; (2) severe colorectal bleeding occurred within 30 days; (3) participating in other clinical trials requiring drugs within 60 days; (4) serious cardiovascular diseases, uncontrolled infections, or other uncontrolled co-existing diseases; (5) pregnant women; (6) subjects receiving preoperative neo-adjuvant therapies and/or radiotherapy. Each patient signed the informed consent, which outlined the purpose and application of the colorectal tumor tissue that they donated. All samples were fixed with 4% paraformaldehyde and cryoprotected in 30% sucrose for immunohistochemical staining or were kept frozen at -80°C for other biochemistry analysis.

### DNA isolation, Polymerase Chain Reaction (PCR) and sequencing

Genomic DNA was isolated from the CRC tissue specimens with a DNA isolation kit (GIAGEN, Hilden, Germany). To examine Fpr1 SNPs c.289C>A, c.301G>C, c.306T>C, c.546C>A, c.568A>T, c.576T>C>G, and c.1037C>A, primers were designed to amplify a 1538bp fragment of the FPR1 gene containing these SNPs. The forward primer was 5'-TTGCCCAACAGGTACAATAA-3' and the reverse primer was 5'-ATTTCAGGCAAACTAGGATG-3'. The products were sequenced with the primers 5'-AGAACCACCGCACCGTGA-3' and 5'-GGATGTTCCGGCTGTTGT-3'. PCR was performed according to the following conditions: 95 ºC for 3 min, 40 cycles of denaturation at 95 ºC for 20 s, annealing at 56 ºC for 45 s, and extension at 72 ºC for 20 s with a final extension at 72 ºC for 10 min. All samples yielded PCR products, which were sequenced by Shanghai DNA biotechnologies.

### Immunohistochemistry

The primary antibodies were mouse anti-human P53 protein and anti-human Ki67 protein (1:50; Santa Cruz Biotechnology, Santa Cruz, CA, United States), and rabbit anti-human CEA protein and rabbit monoclonal anti-HER2 protein (1:100; IBL, Tokyo, Japan). After incubation with the primary antibody, the sections were incubated with the secondary antibody and avidin-biotin-peroxidase complex. The slides were counterstained with hematoxylin and eosin. Positivity for P53 and Ki67 were defined as nuclear staining in > 10% of the tumor cell nuclei. Positivity for CEA was > 10% cytoplasmic staining. HER-2 immunostaining was scored as follows: 0-no reactivity or membranous reaction in fewer than 10% of cells, (1+)-faint complete or partial membranous reactivity in more than 10% of cells, (2+)-moderate complete or basolateral membranous reactivity in more than 10% of cells, and (3+)-strong complete or basolateral membranous reactivity in more than 10% of cells. The level of HER2 membranous expression was considered positive if IHC staining was 2+ or 3+. Immunohistochemical reactivity was interpreted blindly by two independent investigators.

### Data analysis

Statistical analysis was performed using the statistic software GraphPad Prism 5 (San Diego, CA). Correlations between Fpr1 SNPs and various clinicopathological characteristics and specific diagnostic markers of CRC were analyzed by the Chi-square test. P values less than 0.05 was considered statistically significant.

## Results

### Basic clinical characteristics of the colorectal cancer patients

In this study, we enrolled 60 Chinese CRC patients, with 30 (50%) male and 30 (50%) female. Their age ranged from 22 to 83 years (mean age, 54.5 years). 36 patients had colon cancer and 24 patients had rectal cancer. All patients had T0-T4 cancers according to the TNM classification. 29 patients were classified as highly or poorly differentiated and 31 as well or moderately differentiated. Ten of these patients had distant metastasis. The basic clinical characteristics of these patients are listed in Table 3.

### Single nucleotide polymorphisms of Fpr1 in Chinese colorectal cancer patients

Seven previously identified Fpr1 SNPs (c.289C>A, c.301G>C, c.306T>C, c.546C>A, c.568A>T, c.576T>C>G, and c.1037C>A) were detected in Chinese CRC patients. The c.306T>C and c.546C>A are synonymous SNPs and other five sites are non-synonymous SNPs. They are all located within exon 2 of the Fpr1 gene. The allele and genotype frequencies of these SNPs identified by sequence analysis of Fpr1 gene are demonstrated in Table 1 and 2. The minor allele frequency (MAF) for c.289A (p.L97M), c.301C (p.V101L), c.306C (p.F102F), c.546A (p.P182P), c.568T (p.R190W), and c.1037A (p.A346E) were 0.115, 0.448, 0.031, 0.500, 0.135, and 0.344 respectively. The allele and genotype frequencies of these SNPs in CRC tissues were not significantly different from that in whole blood cells derived from healthy Chinese subjects. Data of healthy Chinese subjects are from Zhou's reports 24 and the HapMap resource (CHB, Han Chinese in Beijing, and CHS, Southern Han Chinese at https://www.ncbi.nlm.nih.gov/variation/tools/1000genomes/).

### Correlation of Fpr1 SNPs with clinicopathological characteristics

Next, we examined the correlation between Fpr1 SNPs with clinicopathological characteristics which included sex, age, AJCC stage and location of tumor, tumor size, differentiation, lymphatic invasion, distant metastasis, and pathological type. The significance of association between each of the SNPs and clinicopathological characteristics was determined by Chi-square test. Significant associations were observed between genotypes of c.289C>A and distant metastasis (P=0.001, Table 3). Also, significant associations were observed between genotypes of c.306T>C and tumor size (P=0.016, Table 3). Genotypes of c.546C>A was closer to tumor size and lymphatic invasion (P=0.012 and P=0.043, respectively, Table 4). Meanwhile, genotypes of c.1037C>A was related with tumor location and differentiation (P=0.000 and P=0.005, respectively, Table 4). Besides, genotypes of c.576T>C>G was related with pathological type (P=0.000, Table 5).

Furthermore, by using immunohistochemistry, we investigated some specific diagnostic markers of CRC such as P53, CEA, HER2, and Ki-67. The correlation between Fpr1 SNPs with these markers was analyzed (Table 6). The results showed that several Fpr1 SNP positions including c.289 (C>A) and c.576 (G>C>T) were related to the expression of P53 (P=0.004 and P=0.008, respectively), and c.289 (C>A) and c.306T>C to CEA (P=0.03 and P=0.011, respectively, Table 6). Otherwise, genotypes of c.576T>C>G were also associated with HER2 (P=0.001, Table 6). Besides, genotypes of c.289 (C>A) and c.576T>C>G were related with Ki-67 (P=0.034 and P=0.001, respectively, Table 6).

## Discussion

Colorectal cancer, a highly invasive carcinoma, has high rates of venous invasion, lymphatic duct invasion, and lymph node metastasis 25. Metastatic colorectal cancer (mCRC) is the main cause of the death in CRC patients 25. In our previous study, the expression of FPR1 has been identified to be related to tumor serosal invasion in CRC patients. FPR1 activation promoted the migration and invasion of CRC cells 16. In this work, we found the patients with Fpr1 homozygous 289A genotype showed a higher risk to distant metastasis than that with the 289C/C or 289A/C genotypes (P=0.001), suggesting an association between Fpr1 genotype c.289 and the distant metastasis. In recent years, a large number of metastasis-associated genes have been identified. Via genomic DNA microarray and function annotation, Cai et al. reported that multiple SNP-associated genes specific to CRC metastasis were related to adhesion and immunity, such as cell motion, cell-substrate adhesion, regulation of innate immune response, etc 26. Furthermore, many SNP-associated genes were also associated with adhesion- and immune-related signaling pathways, including adherens junction, chemokine signaling pathway, and cytokine-cytokine receptor interaction 26. Otherwise, Lan and colleagues demonstrated that genetic polymorphisms of insulin-like growth factor 1 (IGF-1) and interleukin-8 (IL-8) increased susceptibility to lymph node metastasis in CRC patients 27. All these data suggested that the polymorphisms of those immune-related genes play an important role in the metastasis of CRC.

As we mentioned earlier, a major cause of death in colorectal patients is metastasis 25. Early clinical trials suggest the role for personalized HER2-targeted therapy in mCRC 28. HER2, also known as ERBB2 or proto-oncogene Neu, is a member of the human epidermal growth factor receptor family. Amplification or over-expression of HER2 has been reported to be closely related to the development and progression in certain aggressive types of cancers including breast cancer and CRC 29,30. HER2 is also a potential biomarker guiding chemotherapy in advanced colorectal cancer 31. Our results showed that genotypes of c.576T>C>G were also associated with HER2. Previous studies have found that multiple factors may influence the expression of HER2 32. Since Fpr1 SNPs are closely associated with HER1 expression in CRC patients, further experiments are needed to verify how Fpr1 SNPs regulate the expression of HER2. Besides, the relationship between Fpr1 SNPs and HER2-targeted therapy in mCRC can also be explored.

Multiple studies reported that the genetic polymorphisms are associated with the occurrence and progression of CRC 33,34. In this work, we found the allele and genotype frequencies of Fpr1 SNPs in CRC tissues were not significantly different from that in whole blood cells derived from healthy Chinese subjects. This result suggested that the polymorphisms of Fpr1 may have no effect on the occurrence of CRC. However, Fpr1 genotypes of c.289C>A, c.306T>C, c.546C>A, c.576T>C>G, and c.1037C>A showed significant associations with the tumor size in CRC patients, indicating their potential role in the progression of CRC. The polymorphism of c.576 was also reported to be associated with susceptibility to stomach cancer and periodontitis 21, 22. These results suggest that Fpr1 polymorphisms may be closely related to the occurrence and/or development of gastrointestinal tumors.

CEA, specifically expressed in biliary and gastrointestinal epithelium, is a diagnostic marker for CRC 35. Previous studies reported that the expression of CEA in CRC was related to survival 36. The expression of CEA is frequently higher in poorly differentiated colon cancer 37. In this study, we found that the genotypes of Fpr1 c.289 (C>A) and c.306T>C were highly associated with CEA expression in CRC. Further experiments are needed to verify the mechanism by which Fpr1 SNPs regulates CEA expression.

Our previous study found that the expression of FPR1 was related to tumor serosal invasion in CRC patients. FPR1 activation promoted the migration and invasion of CRC cells 16. In this work, we further identified that Fpr1 SNP was associated with the distant metastasis of CRC. Fpr1 SNPs were also associated with the expression of P53, CEA, HER2 and Ki67. This is the first study to evaluate polymorphisms of Fpr1 in CRC and find positive correlations. Further replication study with a larger sample size is required. Additionally, research on interaction mechanism between Fpr1 SNPs and the positive related factors is needed.

## Figures and Tables

**Table 1:** Allele frequencies in the CRC patients studied

Reference ID	SNP	Allele	Phenotype	Frequency			
				Chinese CRC patients	CHB* (Han Chinese in Beijing)	CHS* (Southern Han Chinese)	CH^#^ (Han Chinese)
rs78488639	L97M	c.289C c.289A	Leu^97^ Met^97^	0.885 0.115	0.918 0.082	0.938 0.062	None
rs2070745	V101L	c.301G c.301C	Val^101^ Leu^101^	0.552 0.448	0.549 0.451	0.529 0.471	0.550 0.450
rs28930680	F102F	c.306T c.306C	Phe^102^ Phe^102^	0.969 0.031	0.971 0.029	0.981 0.019	None
rs2070746	P182P	c.546C c.546A	Pro^182^ Pro^182^	0.500 0.500	0.524 0.476	0.486 0.514	None
rs5030880	R190W	c.568A c.568T	Arg^190^ Trp^190^	0.865 0.135	0.791 0.209	0.824 0.176	0.828 0.172
rs1042229	N192K	c.576T c.576G c.576C	Asn^192^ Lys^192^ Asn^192^	0.521 0.344 0.135	0.524 0.262 0.214	0.567 0.262 0.171	0.514 0.304 0.182
rs867228	A346E	c.1037C c.1037A	Ala^346^ Glu^346^	0.656 0.344	0.714 0.286	0.705 0.295	0.703 0.297

*Data from the HapMap resource (https://www.ncbi.nlm.nih.gov/variation/tools/1000genomes/); ^#^Data from Zhou's report 24.

**Table 2:** Genotype frequencies in the CRC patients studied

Reference ID	SNP	Genotype	Frequency	
			Chinese CRC patients	CH^#^ (Han Chinese)
rs78488639	L97M	L/L L/M M/M	0.792 0.187 0.021	None
rs2070745	V101L	V/V V/L L/L	0.271 0.562 0.167	0.297 0.507 0.196
rs28930680	F102F	F/F	1.000	None
rs2070746	P182P	P/P	1.000	None
rs5030880	R190W	R/R R/W W/W	0.729 0.271 0.000	0.689 0.278 0.033
rs1042229	N192K	N/N N/K K/K	0.458 0.396 0.146	0.469 0.455 0.077
rs867228	A346E	A/A A/E E/E	0.396 0.521 0.083	0.507 0.392 0.100

^#^Data from Zhou's report 24.

**Table 3:** *Fpr1* SNPs with clinicopathological characteristics (c.289, c.301, and c.306)

Parameters	Mutation of 289			*P* value	Mutation of 301			*P* value	Mutation of 306			*p* value
	C/C	C/A	A/A		G/G	G/C	C/C		T/T	T/C	C/C	
**Gender**												
Female	25	4	1	0.710	4	22	4	0.008	26	4	0	1.000
Male	23	4	3		11	10	9		27	3	0	
**Age (years)**												
≤ 60	26	4	2	1.000	5	22	5	0.042	29	3	0	0.695
> 60	22	4	2		10	10	8		24	4	0	
**AJCC**												
0-II	24	6	0	0.057	6	18	6	0.621	23	7	0	0.011
III-IV	24	2	4		9	14	7		30	0	0	
**Tumor location**												
Colon	26	7	3	0.170	7	21	8	0.474	30	6	0	0.225
Rectum	22	1	1		8	11	5		23	1	0	
**Size of tumor**												
≤ 5	29	5	0	0.064	6	24	4	0.198	27	7	0	0.016
> 5	19	3	4		9	18	9		26	0	0	
**Differentiation**												
I-II	25	2	4	0.057	4	23	4	0.004	27	4	0	1.000
III-IV	23	6	0		11	9	9		26	3	0	
**Lymphatic invasion**												
Yes	21	6	1	0.202	5	19	4	0.108	24	4	0	0.695
No	27	2	3		10	13	9		29	3	0	
**Distant Metastasis**												
Yes	5	1	4	0.001	2	8	0	0.121	7	3	0	0.083
No	43	7	0		13	24	13		46	4	0	
**Pathological Type**												
Adenocarcinoma	35	6	2	0.659	8	25	10	0.222	39	4	0	0.393
Mucinous	13	2	2		7	7	3		14	3	0	

**Table 4:** *Fpr1* SNPs with clinicopathological characteristics (c.546, c.568, and c.1037)

Parameters	Mutation of 546			*P* value	Mutation of 568			*p* value	Mutation of 1037			*p* value
	C/C	C/A	A/A		G/G	G/C	C/C		T/T	T/C	C/C	
**Gender**												
Female	7	15	8	1.000	20	10	0	1.000	11	15	4	1.000
Male	8	15	7		21	9	0		12	14	4	
**Age (years)**												
≤ 60	5	17	10	0.206	27	5	0	0.006	15	12	5	0.218
> 60	10	13	5		14	14	0		8	17	3	
**AJCC**												
0-II	9	12	9	0.283	21	9	0	1.000	10	17	3	0.514
III-IV	6	18	6		20	10	0		13	12	5	
**Tumor location**												
Colon	8	18	10	0.742	24	12	0	0.784	19	17	0	0.000
Rectum	7	12	5		17	7	0		4	12	8	
**Size of tumor**												
≤ 5	8	22	4	0.012	32	2	0	0.000	12	18	4	0.716
> 5	7	8	11		9	17	0		11	11	4	
**Differentiation**												
I-II	6	20	5	0.061	29	2	0	0.000	13	18	0	0.005
III-IV	9	10	10		12	17	0		10	11	8	
**Lymphatic invasion**												
Yes	7	18	3	0.043	20	8	0	0.782	12	12	4	0.724
No	8	12	12		21	11	0		11	17	4	
**Distant Metastasis**												
Yes	4	4	2	0.550	7	3	0	1.000	6	4	0	0.241
No	11	26	13		34	16	0		17	25	8	
**Pathological Type**												
Adenocarcinoma	11	23	9	0.562	36	7	0	0.000	19	20	4	0.208
Mucinous	4	7	6		5	12	0		4	9	4	

**Table 5:** *Fpr1* SNPs with clinicopathological characteristics (c.576)

Parameters	Mutation of 576 n (%)						*p* value
	T/T	T/C	T/G	C/C	C/G	G/G	
**Gender**							
Female	9	6	9	0	4	2	0.463
Male	7	5	9	0	2	7	
**Age (years)**							
≤ 60	12	2	10	0	4	4	0.053
> 60	4	9	8	0	2	5	
**AJCC**							
0-II	9	5	6	0	3	7	0.282
III-IV	7	6	12	0	3	2	
**Tumor location**							
Colon	9	7	12	0	3	5	0.937
Rectum	7	4	6	0	3	4	
**Size of tumor**							
≤ 5	8	6	12	0	2	6	0.628
> 5	8	5	6	0	4	3	
**Differentiation**							
I-II	9	6	9	0	3	4	0.982
III-IV	7	5	9	0	3	5	
**Lymphatic invasion**							
Yes	6	6	10	0	3	3	0.734
No	10	5	8	0	3	6	
**Distant Metastasis**							
Yes	2	2	2	0	2	2	0.688
No	14	9	16	0	4	7	
**Pathological Type**							
Adenocarcinoma	14	9	5	0	6	9	0.000
Mucinous	2	2	13	0	0	0	

**Table 6:** Fpr1 SNPs with specific diagnostic markers of CRC

Parameters (Total mutations)	P53		*p* value	CEA		*p* value	HER-2		*p* value	Ki-67		*p* value
	Yes	No		Yes	No		Yes	No		Yes	No	
**c.289**			0.004			0.030			0.001			0.034
C/C	39	9		43	5		30	18		30	18	
C/A	3	5		5	3		0	8		8	0	
A/A	1	3		2	2		1	3		4	0	
**c.301**			0.409			0.429			0.277			0.060
G/G	9	6		11	4		5	10		14	1	
G/C	25	7		28	4		18	14		20	12	
C/C	9	4		11	2		8	5		8	5	
**c.306**			0.092			0.011			0.247			1.000
T/T	40	13		47	6		29	24		37	16	
T/C	3	4		3	4		2	5		5	2	
C/C	0	0		0	0		0	0		0	0	
**c.546**			0.188			0.283			0.006			0.090
C/C	11	4		12	3		13	2		7	8	
C/A	24	6		27	3		13	17		24	6	
A/A	8	7		11	4		5	10		11	4	
**c.568**			1.000			0.711			0.100			1.000
G/G	29	12		35	6		18	23		29	12	
G/C	14	5		15	4		13	6		13	6	
C/C	0	0		0	0		0	0		0	0	
**c.1037**			0.851			0.714			0.936			0.676
T/T	17	6		19	4		11	12		15	8	
T/C	21	8		25	4		16	13		22	7	
C/C	5	3		6	2		4	4		5	3	
**c.576**			0.008			0.557			0.001			0.011
T/T	7	9		11	5		5	11		13	3	
T/C	10	1		10	1		8	3		10	1	
T/G	14	4		16	2		5	13		14	4	
C/C	0	0		0	0		0	0		0	0	
C/G	3	3		5	1		4	2		2	4	
G/G	9	0		8	1		9	0		3	6	

## Abbreviations

FPR1: formyl peptide receptor 1
CRC: colorectal cancer
SNP: single nucleotide polymorphisms
CEA: carcino-embryonic antigen
